# Techno-economic assessment of a central grid-connected wind farm in Ghana using RETScreen® Expert

**DOI:** 10.1016/j.heliyon.2023.e12902

**Published:** 2023-01-10

**Authors:** Samuel Sarpong Asamoah, Joseph Parbey, Isaac Kwasi Yankey, Alfred Awuah

**Affiliations:** a Department of Energy Systems Engineering, Faculty of Engineering, Koforidua Technical University, Ghana; b Industrial Chemistry Section, Department of Chemistry, School of Physical Sciences, College of Agriculture and Natural Sciences, University of Cape Coast, Ghana; c Department of Environmental Management and Technology, Faculty of Built and Natural Resources, Koforidua Technical University, Ghana

**Keywords:** Net present value, Internal rate of return, Electricity production cost, Simple payback, RETScreen Expert, Greenhouse gas emissions

## Abstract

This paper presents the technical, financial, and environmental impact assessment of a 50-MW (MW) utility-scale wind farm in Ghana at four locations: Anloga, Atiteti, Sege, and Denu. The monthly average wind speeds recorded at the locations were 6.01 m/s, 5.98 m/s, 5.46 m/s, and 5.17 m/s respectively at 60 m above ground level. Capacity factors of 24.9%, 24.4%, 20.6%, and 18.0% were obtained at the locations respectively. The Net Present Value (NPV) was the main financial metric employed to determine the viability of the projects. The results indicated that a potential utility-scale wind project is viable at all locations under study. Furthermore, the Electricity Exported to the Grid and the Electricity Export Rate (EER) had the highest impact of 0.63 and 0.62 on the NPV respectively and therefore these key parameters should be well considered before any potential project implementation.

## 1 Introduction

Year after year, the negative impacts of climate change become increasingly apparent coupled with worldwide energy-related CO_2_ emissions which is rising by 1% per year on average over the previous decade, although sometimes leveling down [1,2]. The long-term supply and use of fossil-based fuels have led to this climate crisis. In addition, its continuously escalating costs and their inevitable depletion arises another issue of global energy security [3]. There is a lot of interest because of recent incentives to minimize greenhouse gas emissions especially carbon dioxide, which has increased the pursuit of alternative and sustainable energies [3]. The International Renewable Energy Agency (IRENA) asserts that, “The energy transition is already taking place, and it is unstoppable” [4]. In the last decade, significant progress has been accomplished, with governments and markets favoring renewable-based energy sources, especially the financial sector [4].

It is critical for Africa and the world's economic and energy futures to fulfill the energy demands of a young, rapidly rising, and expanding urban population. Furthermore, the International Energy Agency (IEA) claims that, nearly one-in-two persons added to the global population between now and 2040 will be Africans; and by 2025, Africa's population would outnumber both India and China [3]. As a result of this circumstance, it is expected that renewable energies including wind power will play a key part in attaining a low-carbon or – in many markets – net-zero future. This will yield a carbon-free energy industry as well as significant reductions in emissions across the global economy [5]. In addition, the Global Wind Energy Council (GWEC) stated that 60.4 GW of new wind power plants were installed in 2019 globally, raising the overall capacity of wind energy to 651 GW. In the onshore market, 54.2 GW was installed, representing a 17% growth over 2018 [5]. China and the United States were the world's major onshore markets, accounting for more than 60% of new onshore additions between them [5]. However, Africa's share of new onshore wind energy installations was 0.94 GW in the same year [5]. Ghana's annual electricity consumption growth is anticipated to be around 10% [6–8]. In the medium to long future, the Ghanaian Energy Commission anticipates that an electrical capacity gain of roughly 200 MW per year will be necessary to keep up with the rising demand [6,7]. In addition, electricity demand was expected to reach over 23,000 GWh by 2020, growing to around 40,000 GWh by 2030 [6,8].

The effects of climate change on energy demand and supply and renewable energy resources have been studied by Refs. [[9], [10], [11], [12]]. For the correct and advantageous establishment of a wind farm at any location, wind resource data analysis and precise wind power calculation are required. Studies have established that climate change affects wind speeds globally [13,14]. There is however uncertainty about the effect of climate change on wind energy generation compared with other renewable energy systems. For instance, Gernaat et al. [9] reported both increases and decreases in wind energy resources using integrated climate models to estimate effects on renewable energy resources. Eichelberger et al. [13] and Harvey [14] reported that climate change has led to an observed decrease in wind speeds and may affect wind power output [13,14]. Furthermore, the Intergovernmental Panel on Climate Change (IPCC) cited by Robbins [15], affirms wind speed will drop over the upcoming decades. The IPCC estimates that by the year 2100, the average annual wind speeds could decrease by up to 10% due to climate change.

El Khchine et al. [16] evaluated the wind potential and trends in some regions in Morocco. The Weibull parameters were estimated using mean hourly wind speed data while incorporating several approaches such as standard deviation, wind variability, power density, Moroccan, and WAsP methods. Their annual shape parameter varied from 1.65 to 4.85 with its annual scale parameter ranging from 4.05 m/s to 10.03 m/s, making the locations under study suitable for utility-scale power generation.

El Sattar et al. [17] economically evaluated wind energy in Egypt considering the Levelized Cost of Energy in some regions in Egypt. They established that a potential wind farm implementation in two regions were economically feasible. The LCOE ranged from 0.052 to 0.326 $/kWh for three regions that were under study with an average capacity factor range of 23.5–58%.

Rafique et al. [18] conducted a feasibility of a 100 MW wind farm at different locations in Saudi Arabia. They concluded that the proposed wind power farm was viable both technically and economically with all sites estimated to be profitable.

Himiri et al. [19] estimated the wind power potential at three (3) locations in Algeria. The estimated capacity factors of the wind farm were in the range of 21%–38% with positive NPVs at all locations.

Adnan et al. [20] evaluated wind energy production in Pakistan by analyzing mean wind speeds and Weibull parameters. They obtained an Electricity Production Cost of $0.074/kWh and $0.056/kWh for two (2) locations with an estimated payback period of 7 years.

Charabi and Abdul-Wahab [21] assessed the performance of a wind turbine for electricity cost minimization in Oman using HOMER Pro software. An Electricity Production Cost of $0.171/kWh and $0.070/kWh was obtained for various locations in the country.

Moya et al. [22] assessed geothermal energy in Ecuador using RETSceen software. After considering three scenarios of incentives for the geothermal power plant, all scenarios had a positive NPV except scenario IIIA. They obtained equity paybacks of 3.2, 3.7, 16, and 5.6 years for the Scenarios under study. In addition, they estimated that about 184,000tCO_2_ could be saved annually by eliminating fossil fuel power production.

Kofi et al. [23] assessed a hybrid system comprising a solar/diesel system using RETSreen software in Ghana. They came out with an optimal hybrid PV-DG grid-connected system for J. A. Plant Pool Ghana Limited for its warehouse department. In addition, they conducted an economic evaluation of the project, revealing that the project was viable. Furthermore, both the annual earnings from electricity exports to the grid and the reduction in GHG emissions from the project fell within the established criteria.

Ahmed et al. [24] (2021) conducted a techno-economic evaluation of single-end-energy users to reduce GHG emissions in Pakistan using MATLAB, Helioscope, and RETSreen software. They revealed that per annum, the end energy user will save about 3570.6 L of gasoline when they switch to a clean energy source using a rooftop PV system. Additionally, they estimated that the project investment could be recouped in 5 years (equity) for a grid-tied investment of $7337; and 9 years (equity) for a standalone investment which was estimated to be $9077 for 25 years.

Samuel [25] 2021 evaluated a small-scale hydropower project for rural electrification in Karela, India. The Electricity Production Cost of the project was 1.501 INR/kWh, a cost-benefit ratio of 4.6, an NPV of INR991,160,233, and, a simple payback of 5.9 years. He concluded that the project was economically viable with high profits because its NPV was greater compared to its initial cost.

Thi Thi Soe et al. [26] (2015) conducted an economic assessment of some promising wind energy sites in Myanmar, China. They estimated that a proposed wind farm of 25 kW comprising 24 units yielded 0.148$/kWh, 0.141$/kWh, 0.171$/kWh and 0.138$/kWh for the four locations which were assessed. This was economically viable compared to the price of a diesel generator which was 0.3$/kWh. In addition, the project yielded positive NPVs of $251.753, $247.688, $248.252, and $251.699 respectively.

Mostafaeipour et al. [27] (2020) conducted a statistical analysis of using new wind generators in South Africa. The results of their research indicated that when utilizing the EOLO wind turbine, Port Elizabeth station had the lowest Levelized cost of energy (LCOE) at 0.363$/kWh, while Bloemfontein's facility recorded the highest LCOE of 1.601$/kWh utilizing a Turby wind turbine.

Almutairi et al. [28] conducted a ranking of some places for hydrogen production utilizing wind and solar hybrid energy in 2021. The rankings indicated that population, ambient temperature, altitude, relative humidity, price of land, skilled labour, facilities, topographic condition, and distance from main roads were all shown to affect hydrogen generation, as were wind and solar energy. In addition, the cities which were studied were ranked utilizing the additive ratio assessment (ARAS), weighted sum method (WSM), and weighted aggregated sum product assessment (WASPAS) techniques. The results indicated that East London and Bloemfontein were found to be the most and least suitable stations, respectively, for the use of domestic-scale wind turbines.

Sahri et al. [29] evaluated of an energy management system for a hybrid PV/Wind/Battery/Fuel Cell in a micro-grid Hydrogen and an economic evaluation of a hybrid/Battery Supercapacitor Energy Storage. They assessed the potential of using excess wind energy to create and store hydrogen, and when there was insufficient wind energy, a solid oxide fuel cell system (SOFC) was used to renew electricity using the stored hydrogen. A power management algorithm was also employed to moderate fluctuations caused by the changes in the wind speed, prevent overcharging and deep battery discharge, prevent battery overheating, and meet its load profile requirements.

In mitigating climate change and its impact on the globe, Paletot et al. [30] assessed the impact of biomass plants on the environment to enhance the social tolerance of renewables in Italy using a Life Cycle Approach. The results indicated that an average climate change effect of bioenergy plants of 45.84 gCO_2eq_ MJ^−1^ and a range between 14.93 gCO_2eq_ MJ^−1^ and 90.70 gCO_2eq_ MJ^−1^ was obtained.

Neto [31] evaluated the impact of municipal solid waste on a sizable-scale plant for the largest metropolitan region in Brazil. According to the input and output models adopted, there was a decrease in energy demand of −0.31% with a −3.40% reduction in GHG emissions.

Adebayo et al. [32] assessed how renewable energy influences carbon emissions which are based on consumption in MINT (Mexico, Indonesia, Nigeria, and Turkey) economies. They established that both common correlated effect mean group (CCEMG) and the augmented mean group (AMG) showed that while globalization and the use of renewable energy help to slow down environmental deterioration, economic expansion and the use of nonrenewable energy contribute to it. The results of the causality test also demonstrated the ability of all regressors to forecast CO_2_ emissions in the MINT nations.

Misila et al. [33] assessed Thailands's achievement on renewable energy (RE) and energy efficiency (EE) in GHG emission reduction for the country in the long term. It was established that the potential of domestic RE and EE initiatives to realize Thailand's nationally decided contribution is one of the outcomes (NDC). Furthermore, it was discovered that both objectives in the RE plan and the EE plan must be accomplished by at least 50% and 75%, respectively, to reach Thailand's first NDC's 20% GHG emission reduction target in 2030. Alternatively, targets in the RE plan and EE plan must be accomplished by at least 75% and 50%, respectively.

Mulopo [34] (2022) sought to examine the last ten years' worth of effective renewable energy efforts in Africa (2010–2020). Her results implied that Sub-Saharan African interventions in renewable energy are founded on established and commercialized technology and that natural resources for power production have not yet been exploited fully. In addition, her results indicated the existence of off-grid technology interventions coupled with the necessity of integrated project design and execution in Sub-Saharan Africa, which takes into account the adoption of a decentralized strategy for renewable energy generation, particularly in rural regions, as well as factors like community engagement, social, economic, institutional, and technical participation, as well as have all hampered the growth of renewable energy sources.

In Ghana, the wind resource is greatest near the coast east of the Greenwich Meridian [8]. In addition, wind speeds observed at 60 m and 80 m heights at sixteen (16) places around the country ranged between 4.0 m/s and 7.0 m/s [6]. It is estimated that this level of wind speed is sufficient to generate utility-scale power with current technology [6,11,16]. Moreover, the projected capacity factor of wind farms in Ghana is estimated to be above 20% [35]. Despite this potential, the country is yet to develop a utility-scale wind farm to augment its electricity demand [16,17]. However, the country currently has a few downstream firms in the wind power industry value chain, most marketers of small wind power systems, and a few developers. Currently, Ghana is bedeviled with a multitude of energy problems including overcapacity, relatively expensive tariffs (considering the income levels of the majority of the Ghanaian population), and a relatively high percentage of transmission and distribution losses. According to the nation's Renewable Energy Master Plan, renewable energy will account for 1363.63 MW of the nation's total power generation by 2030, up from 42.5 MW in 2015 [6]. Its primary policy objective is to invest in clean energy technology and enhance power generation while enhancing energy efficiency [6]. Ghana's current electricity installed capacity was at 5082.50 MW as of June 2020, with renewable energy accounting for less than 1% [37,38]. One factor contributing to this predicament is a long-standing lack of interest in the integration of mini-grid and utility-scale power generation that will make optimal use of tiny hydropower sites, solar resources, wind resources, and biomass resources across the country. There is currently little knowledge available regarding Ghana's central-grid-connected wind farms' financial feasibility and investors have expressed reservations regarding the economic feasibility of Ghana's wind energy industry. This research will conduct a detailed assessment of the technical and financial viability of a utility-scale wind power generation using RETScreen® Expert for four locations along the coastal belt of Ghana to serve as information for potential investors. The software's wind energy model was chosen for the locations in this research because it places a strong emphasis on the characteristics of the wind turbine matching with wind data from the locations and also provides a detailed economic analysis for a project decision.

## 2 Location and wind resources

**Fig. 1:**
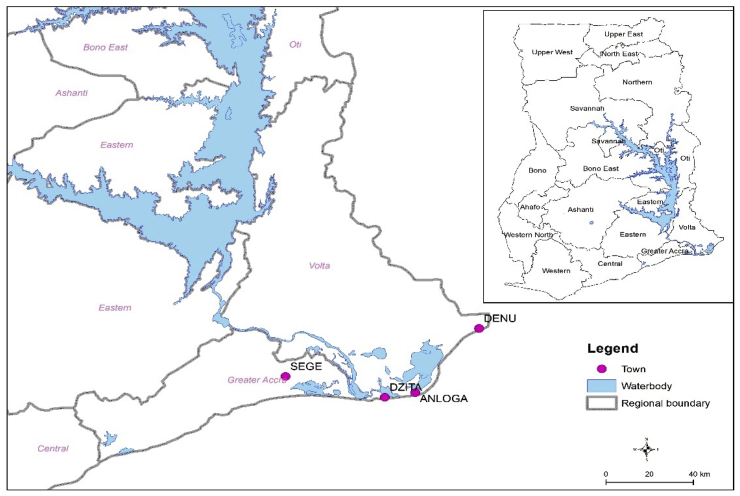
Map of the four locations.

Wind measurement was undertaken by the Ghana Energy Commission in conjunction with GEDAP/MoE (World Bank) spanning the years 2011–2013 for some coastal locations in Ghana [39]. Four locations were selected for analysis based on the highest wind speed to determine the financial viability of a potential central grid-connected wind farm. The locations are Anloga, Atiteti/Dzita, Sege/Ningo, and Denu which are located in the Volta Region of Ghana except for Sege; which is in the Greater Accra Region which is shown in Fig. 1.

The majority of the people in these areas work in the agricultural, forestry, and fishing industries. Furthermore, these regions are low-lying coastal plains with a maximum elevation of 53 m above sea level and a minimum elevation of 1–3.5 m below sea level, rendering them vulnerable to tidal surges and sea erosion [40]. The wind resources obtained at the locations were ground-measured data which was obtained from the Energy Commission of Ghana. The latitude, longitude, altitude, measurement years, and monthly average wind speeds are summarized in Table 1.

**Table 1:** Geographical description and average monthly wind speed of the locations used [39].

Location	Latitude	Longitude	Altitude (m)	Wind speed (m/s)	Measurement years
Anloga	5.787°N	0.919°E	5	6.01	Dec 2012–Dec 2013
Atiteti/Dzita	5.774°N	0.714°E	13	5.98	Dec 2011–Dec 2013
Sege/Ningo	5.872°N	0.345°E	21	5.46	Nov 2011–Dec 2013
Denu	6.112°N	1.141°E	8	5.17	Dec 2012–Dec 2013

**Fig. 2:**
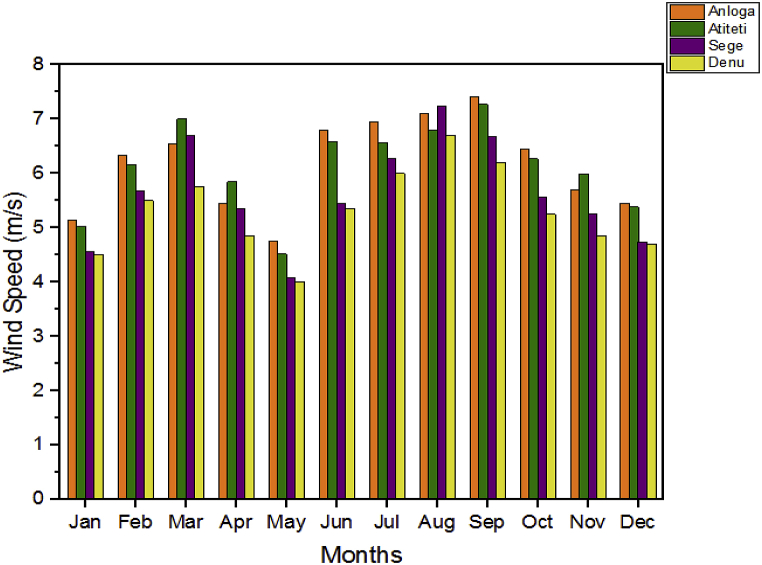
Monthly wind speeds at the four locations [39].

The variations in the monthly average wind speed during the two-year period for the selected locations used for the study are shown in Fig. 2. The figure indicates Anloga has the highest average monthly wind speed throughout the year except for March, April, August, and November with Denu recording the lowest wind speed all year. Also, Anloga obtained the highest monthly average wind speed of 6.01 m/s followed by Atiteti/Dzita (5.98 m/s), Sege/Ningo (5.46 m/s), and Denu (5.17 m/s) in that order.

### 2.1 Limitation

Turbine micro-siting was not considered together with other activities that encompass a typical feasibility study of a wind farm construction in this study. However, the wind resource data used in this study were obtained from a feasibility study report [39]. Furthermore, all costs associated with a typical wind farm project implementation were taken into consideration in performing the financial assessment presented in this paper.

## 3 Methodology

### 3.1 Wind power estimation

**Fig. 3:**
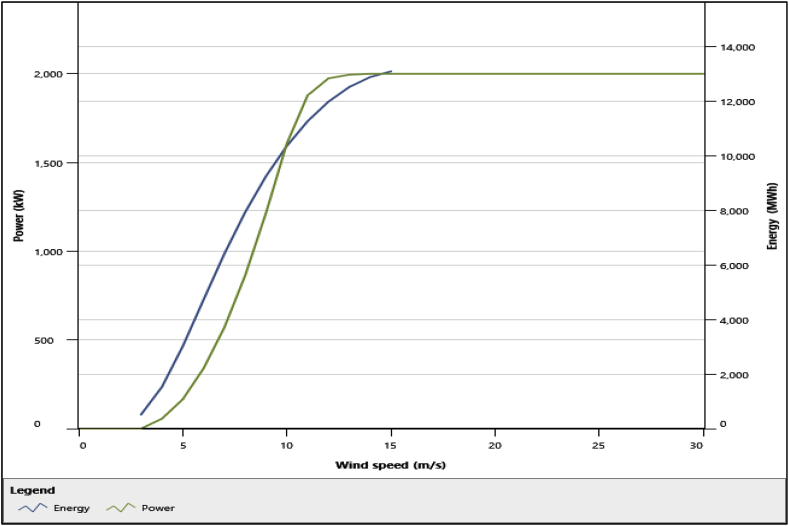
Power and Energy curves of Vestas V90 [41].

The wind turbine chosen for analysis was *VESTAS V90-2.*0 MW *- 80 m* which was obtained from RETScreen® Expert's wind turbine database. Its power production was determined by the software using Eq. (1). The hub height for this turbine was 80 m and its lifetime was assumed to be 20 years. A total of 25 turbines producing 50 MW was adopted for each location and used for analysis. Technical losses of 7%, 5%, and, 3% were considered for array losses, airfoil losses, and miscellaneous losses respectively. Detailed features of the selected wind turbine and its power and energy curves are shown in Table 2 and Fig. 3 respectively.

**Table 2:** Technical characteristics of vestas V90 [41].

Turbine	Value
Manufacturer	Vestas
Model	VESTAS V90-2.0 MW – 80 m
Power Capacity per Turbine	2.0 MW
Number of Turbines	25
Hub Height	80 m
Rotor Diameter per turbine	90 m
Swept area per turbine	6361.73 m^2^
Cut-in-wind speed	4.0 m/s
Rated wind speed	13.0 m/s
Cut-out-wind speed	25.0 m/s
Rotor speed	14.9 rpm
Tower type	Tubular

The power from a wind turbine is calculated by Eq. (1) [41];

(1) $$P_{WTG}=\left(\frac{\rho }{\rho_{o}}\right).P_{WTG,STP}$$

where P_WTG_ is the wind turbine's power output, P_WTG,STP_ is the power output in kW of the wind turbine at the selected location at standard temperature and pressure, ρ is the air's real density in kg/m^3^ and ρ_o_ is the standard absolute temperature at 288.3 K and pressure at 101.3 kpa.

### 3.2 Technique for extrapolating wind speed data

The power law is an equation that uses wind speed measurements at lower altitudes to anticipate wind speed at higher altitudes. Data on wind speed may be extrapolated using this technique. In Eq. (2), the power law as described by Ohunakin et al. [42] is mathematically specified.

(2) $$\frac{U_{h}}{U_{ho}}={\left(\frac{h}{h_{o}}\right)}^{\alpha }$$

where, h is the elevation at which wind speed U_h_ should be calculated; h_0_ is the height at which wind speed U_ho_ was determined, and α is the wind shear exponent.

In Eq. (3), Kocak [43] presents a mathematical connection to calculate the wind shear exponent:

(3) $$\alpha =\frac{0.37-0.088{\;In\;U}_{h_{o}}}{1-0.088\;In\left(\frac{h_{o}}{10}\right)}$$

where: U_ho_ = measured wind speed, h_o_ = Height of anemometer.

### 3.3 Economic feasibility assessment

The economic assessment was performed at the four locations using the RETScreen® Expert Software, which can perform detailed analysis using the given inputs which is presented in Table 4. The cost per kW of the turbine was obtained from the global weighted-average total installed cost of onshore wind turbines in Africa for 2019, which was approximately $2000/kW [44]. In addition, the operation and maintenance (O&M) cost for onshore wind plants globally ranged from $33/kW per year (in Denmark) to USD 56 USD/kW per year (in Germany) [44]. Therefore, an average cost of $45/kW was used as input for the O&M cost in this research. The economic performance of system metrics and its definitions that were used by the RETScreen® Expert Software for the detailed economic analysis are defined as follows:

**Table 3:** Summary of technical assessment.

Location	Anloga	Atiteti	Sege	Denu
Capacity Factor (%)	24.9	24.4	20.6	18.0
Annual Electricity Exported to the grid (MWh) Excl. Tech. Loses	122,232	119,513	122,232	100,801
Annual Electricity Exported to the grid (MWh) Incl. Tech. Loses	109,209	106,780	90,062	78,736

#### 3.3.1 The Net Present Value (NPV)

*The Net Present Value (NPV)* which represents the discounted cost of all cash flows in this project was determined by Eq. (4) [41]:

(4) $$NPV=\sum_{n=0}^{N}\frac{C_{n}}{{\left(1+R\right)}^{n}}$$

where *r* is the discount rate, C_n_ is the after-tax cash flows and n is the number of years of cash flows.

#### 3.3.2 The internal rate of return (IRR)

*The internal rate of return (IRR)* is the discount rate that results in the project's Net Present Value (NPV) being zero. It is calculated by solving the following formula for the *IRR* using Eq. (5) [41]:

(5) $$0=\sum_{n=0}^{N}\frac{{Ċ}_{n}}{{\left(1+IRR\right)}^{n}}$$

where *N* is the project life in years and Ċ_n_ is the cash flow for year *n.*

#### 3.3.3 Simple payback (SPB)

The simple payback *SPB* is the period it takes for the proposed project to reclaim its initial cost in the form of cash flows or savings it generates. This is calculated by Eq. (6) [41]:

(6) $$SPB=\frac{C-IG}{\left(C_{ener}+C_{capa}+C_{RE}+C_{GHG}\right)-\left(C_{O\&M}+C_{fuel}\right)}$$

where C is the project's total beginning cost, IG is the value of incentives and grants, C_ener_ is the annual energy savings or income, C_capa_ is the annual capacity savings or income, C_RE_ is the annual renewable energy generation credit income, C_GHG_ is the GHG reduction income.

#### 3.3.4 Electricity Production Cost (EPC)

The average cost per kWh of useable electrical energy produced by the system is the Electricity Production Cost (EPC). The program uses this approach to divide the entire annualized cost of producing electricity by the total delivered electric load to determine the EPC [41]:

(7) $$EPC=\frac{C_{ann.tot}-C_{boiler}H_{served}}{E_{served}}$$

where C_ann. tot_ is the overall cost of the system yearly, C_boiler_ is the boiler marginal cost ($/kWh), H_served_ is the entire thermal load served (kWh/yr), E_served_ is the sum of electrical load served.

Since there are no thermal loads to consider in this research, the EPC in Eq. (7) becomes:

(8) $$EPC=\frac{C_{ann.tot}}{E_{served}}$$

Hence, Eq. (8) becomes appropriate to represent the approach to determine the EPC.

## 4 Results & discussion

RETScreen® Expert software was used in this research to determine the feasibility of a potential implementation of a 50 MW grid-connected wind farm in four locations along the coastal belt of Ghana. The study involves a comprehensive technical, financial, GHG emissions, sensitivity, and risk analysis.

### 4.1 Technical assessment

The power law in Eq. (2) was used to extrapolate the recorded wind speed data to a hub height of 80 m using RETScreen software. Natural Resources Canada [41] indicates that when the site features are still being determined, a value of 0.14 is a good first approximation. This was used for extrapolation of the wind speed from 60 m to 80 m. The wind speeds increased to 6.4 m/s, 5.9 m/s, and 5.5 m/s for Anloga, Atiteti, Sege, and Denu respectively.

In addition, the annual gross energy yield for zero percentage losses for the four locations on the installation of the 50 MW wind farm are 122,232 MWh, 119,513 MWh, 122,232 MWh and 100,801 MWh for Anloga, Atiteti, Sege, and Denu, respectively. When technical losses are taken into consideration, the actual energy delivered to the grid were 109,209 MWh, 106,780 MWh, 90,062 MWh, and 78,736 MWh, respectively. A Summary of the technical performance of wind farms is presented in Table 3. Based on the Technical losses assumed, the RETScreen® Expert software calculated the capacity factors of the wind farms, which ranged from 18.0% to 24.9% which are presented in Table 3. Even while technological advancements have increased outputs across the board, resource quality has a substantial influence on capacity factors. The capacity factor yield for the locations was quite low compared to the average global yield of about 36% in 2019 [44]. However related studies conducted by Thi Thi Soe et al. [26] yielded similar capacity factors of 20%–24.9% in 2015. In addition, similar studies from Himri et al. [19] estimated the capacity factors of wind farms in some locations in Algeria to be in the range of 21%–38% while El Satar et al. [17] estimated capacity factors of some wind farms in Egypt to be in the range of 23.5%–58%.

### 4.2 Economic & financial assessment

**Fig. 4:**
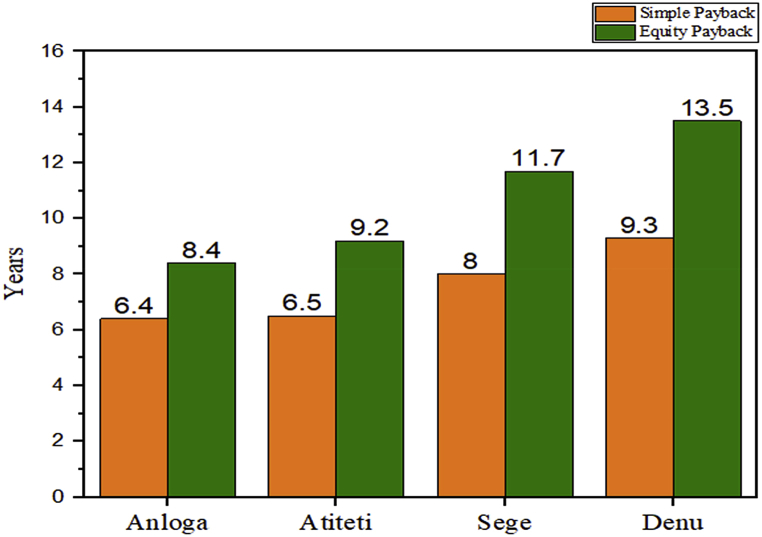
Payback periods at the four locations.

Financial assessment of wind projects is very important before any potential project implementation and this will help determine how economically viable and sustainable the project will become. The financial analysis worksheet of RETScreen® Expert software enables the modeler to input economic parameters that include the rate of inflation, discount rate, debt ratio, debt interest rate, and others which are shown in Table 4. Some of the financial variables that were used were international standards obtained directly from RETScreen® and the International Renewable Energy Agency (IRENA) while others were obtained from local prevailing rates. Details of the financial input variables and their corresponding references that were used in this research are seen in Table 4. The software program calculated the Net Present Value (NPV), the Internal Rate of Return (IRR), the Simple Payback Period (SPB) and the Electricity Production Cost. Furthermore, the yearly life savings, and other financial metrics based on the supplied inputs were also determined by the software which is shown in Table 5. According to Mehmood et al. [45], a project's economic feasibility is determined by its net present value (NPV), internal rate of return (IRR), and payback period. In that regard, this proposed project assessment used the NPV as the main indicator for analysis because it is the main determinant in a potential project decision [46]. As a result, a positive or negative value of the NPV is indicative of whether the project is financially viable or not. Anloga, Atiteti, Sege, and Denu recorded positive NPVs values. Also, the IRR thus; the discount rate that makes the NPV equal to zero was determined for all locations. Anloga had the highest IRR of 19.3%; 18.3%, 12.3%, and 8.4% were also obtained for Atiteti, Sege, and Denu respectively. Furthermore, the simple payback period is the projected cash flow for a particular investment to recover its initial cost which is presented in Fig. 4 at the four locations. The wind farm at Denu yielded the highest payback period of 9.3 years, then 8.0 years for the one at Sege, that of Atiteti was 6.5 years while Anloga yielded the quickest payback period which was 6.4 years. Furthermore, the projects at Sege and Denu yielded equity payback periods of 11.7 and 13.5 years which is an indication that the project will not be able to recoup its debt of 70% of its initial investment at the Bank of Ghana interest rate of 14.5% within the assumed term of 10 years. However, Anloga and Atiteti yielded shorter equity payback periods of 8.4 and 9.2 years which is an indication that its initial investments and debts can be recovered within the debt term that was used. In addition, according to the IRENA, the weighted average Levelized cost of energy for wind projects in Africa is increasingly becoming competitive to large-scale hydro; and this was in the range of $0.050/kWh to $0.072/kWh in the year 2019 [44]. However, the Levelized Cost of Energy or the Electricity Production Cost (EPC) for the various locations was higher compared to the continental values. Anloga yielded the lowest EPC of $0.124/kWh, Atiteti yielded $0.127/kWh, Sege followed with a $0.151/kWh, and Denu with an EPC of $0.173 kWh which is shown in Table 5. However similar studies have shown an LCOE range of 0.041–0.326 $/kWh [17,20,21].

**Table 4:** Financial inputs of RETScreen®.

Financial Inputs	Rate	Reference
Inflation rate	2%	[41]
Discount Rate	5%	[47]
Project Life	20 years	[41]
Debt ratio	70%	Author's Assumption
Debt Interest Rate	14.5%	[48]
Debt term	10 years	Author's Assumption
Electricity Export rate	$0.16/kWh	[49]
Electricity Export Escalation Rate	2%	Author's Assumption
Initial Cost	$2000/kW	[44]
Annual Operation and Maintenance Cost	$45/kW	[44]

**Table 5:** Financial output variables.

Financial Output	Anloga	Atiteti	Sege	Denu
Net Present Value (NPV) ($)	99,433,518	93,455,292	52,315,203	24,444,202
Annual Life cycle savings ($)	7,978,803	7,499,094	4,197,907	1,61,466
Internal Rate of Return (IRR) (%)	19.3	18.3	12.3	8.4
Simple Payback (SPB) (Yr)	6.4	6.5	8.0	9.3
Cost Benefit Ratio	4.3	4.1	2.7	1.8
Electricity Production Cost (EPC) ($/kWh)	0.124	0.127	0.151	0.173

### 4.3 GHG emission reduction analysis

RETSceen® Expert GHG emission worksheet enables the modeler to estimate the GHG emission reduction of a potential project. The following technical considerations were used for the greenhouse gas emission analysis. These considerations are the Transmission & Distribution Loses (T&D) of 22.587% [50] and a GHG Emission Factor of 0.46tCO_2_/MWh which is the factor for wind and solar energy projects in Ghana [36].

**Fig. 5:**
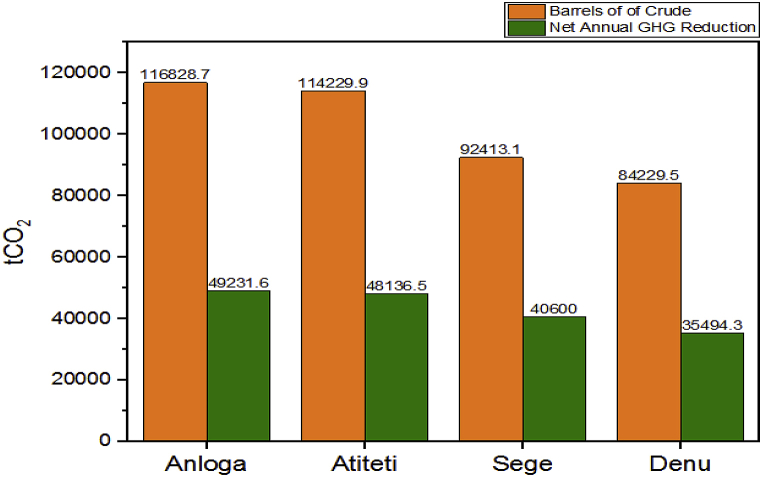
Net GHG emission reduction and its equivalent barrel of crude oil not used.

The software determined the gross annual GHG emission reduction for each location by deducting the proposed case's calculated emission from the based case's calculated emission, as indicated in Table 6. This is because only GHG emissions during the project's operating phase were assessed by the software and not the project's whole life cycle. The wind farm in Anloga yielded the highest GHG emission reduction of 50,236.4 tons of CO_2_ which can be equal to 116,838.7 barrels of light crude oil not consumed which is shown in Table 6 and Fig. 5 respectively. It was followed by the proposed wind farm in Atiteti, Sege while the lowest emission reduction occurred at Denu. The IPCC has determined that fossil fuel emissions are the primary source of global warming, accounting for approximately 89% of worldwide CO_2_ emissions from energy usage [51]. Crude oil usage has declined in recent years due to the advancement of clean energy technologies for global sustainable development. Moreover, countries under the United Nations have committed to achieving the Sustainable Development Goals (SDGs) by 2030. This involves managing the planet's natural resources, combating climate change, creating resilient communities, attaining equitable growth, and eradicating poverty and hunger sustainably while providing energy services. As a result, if this project is implemented, it will aid in the achievement of the given objectives.

**Table 6:** GHG emission reduction for the locations.

Location	Base Case (tCO_2_)	Proposed Case (tCO_2_)	Gross Annual GHG Emission Reduction (tCO_2_)
Anloga	64,737.6	14,501.2	50,236.4
Atiteti	63,540.4	14,331.5	49,118.9
Sege	53,517.7	12,089.1	41,428.6
Denu	46,788.1	10,569.4	36,218.7

Furthermore, the cost of carbon offsets on the carbon trading market is in the range of $1 per ton to $50 per ton [52]. A price of $10 was used in this analysis at an escalation rate of 2% annually. A GHG transaction fee of 2% which is the percentage of credits that has to be paid each year to the crediting agency was also used. Table 7 shows the revenue that will be accrued by selling the GHG emission reduction of the project via carbon trading.

**Table 7:** Net Annual GHG emission reduction and revenue.

Location	Net GHG emission reduction (tCO_2_)	Revenue ($)
Anloga	49,231.7	492,316
Atiteti	48,136.5	481,365
Sege	40,600.0	406,000
Denu	35,494.3	354,943

The program computed the revenue from GHG reduction at the four locations with the wind farm at Anloga generating an annual GHG revenue of $492,316 followed by the wind farm in Atiteti, generating a revenue of $481,365 while the lowest income yield was from the proposed project at Denu. This is because Denu yielded the least level of net GHG emission when compared to the other locations.

### 4.4 Sensitivity analysis

**Fig. 6:**
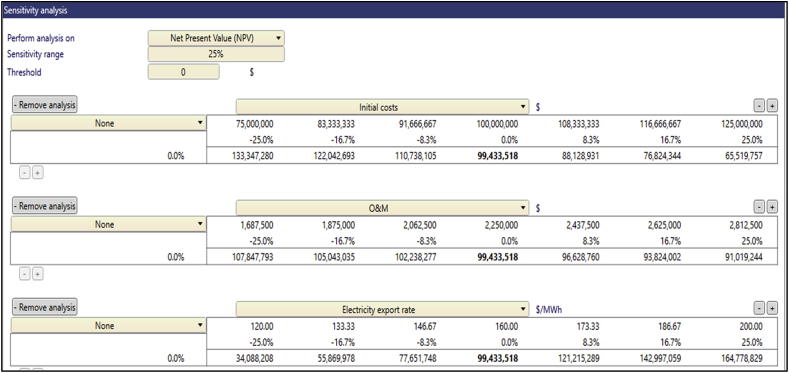
Sensitivity analysis worksheet at Anloga.

**Fig. 7:**
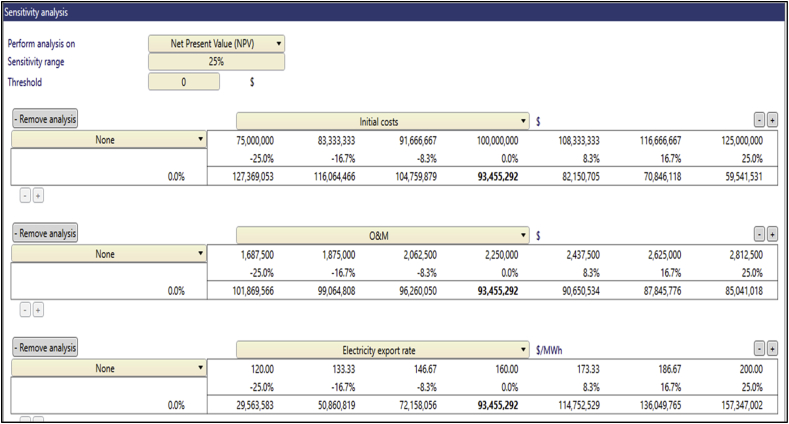
Sensitivity analysis worksheet at Atiteti.

**Fig. 8:**
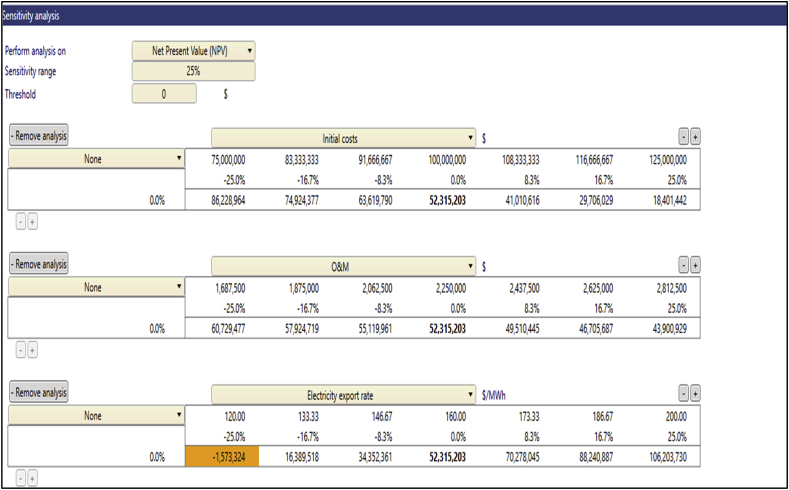
Sensitivity analysis worksheet at Sege.

**Fig. 9:**
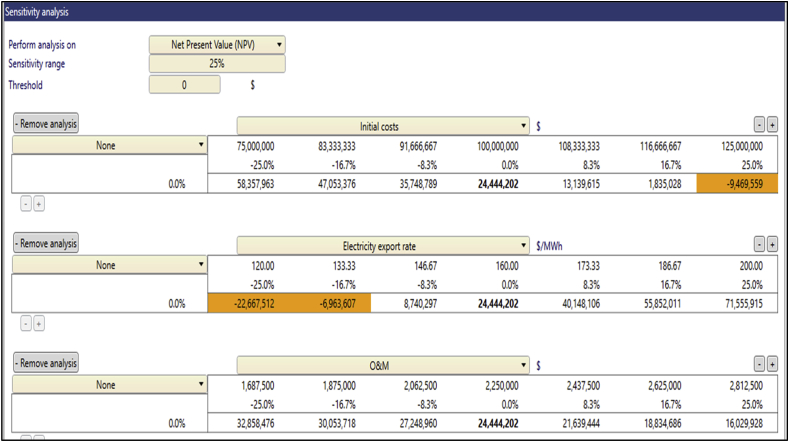
Sensitivity analysis worksheet at Denu.

The sensitivity analysis was performed to determine the level of uncertainties of the financial variables such as inputs that affect the economic metrics of the project. This analysis was performed on financial variables including the Electricity Export Rate, Initial Cost, O&M cost, and Debt Interest Rate. As previously mentioned, the main financial metric that was analyzed was the NPV, which main factor that determines the viability of a potential project decision [46]. The test range employed in this analysis was ±25% with a 0 threshold on the financial metric. Three key financial inputs thus the Initial Cost, O&M Cost, and the Electricity Export Rate were analyzed for their sensitivity to the NPV. The NPV remained positive at Anloga and Atiteti for all the financial variables that were analyzed above but became negative at Sege when its EER was reduced by 25% as depicted in Fig. 6Fig. 7Fig. 8Fig. 9 respectively. Furthermore, the sensitivity threshold for the NPV at Denu was 25% for Initial Cost and −16.7% for the Electricity Export Rate which is highlighted as shown in Fig. 9.

### 4.5 Risk analysis

**Fig. 10:**
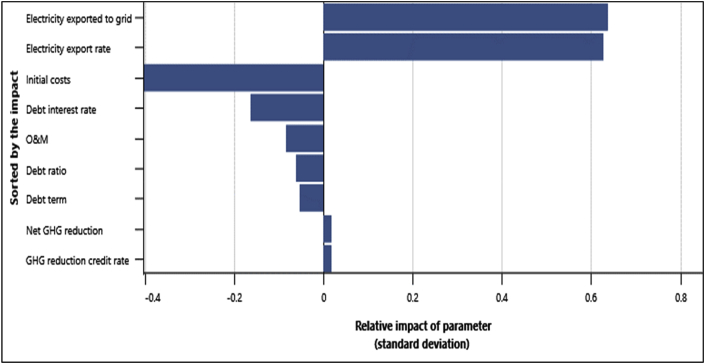
Relative Impact of parameters on the NPV.

This analysis allows the modeler similar to the sensitivity analysis, to determine uncertainties associated with key input parameters of the project. In this analysis, the impact of the input parameters on a financial indicator was determined by using a Monte Carlo simulation that constitutes about 500 to 5000 values of the financial indicator. This is done to determine whether the variation in the financial indicator is acceptable or not by the distribution of the outcomes. Key input variables were tested in the range of ±25% with the Monte Carlo simulation as depicted in Fig. 10. From the impact graph, the electricity exported to the grid and the electricity export rate are variables that had the highest impact on the NPV with an impact of 0.64 and 0.63 respectively and this was the same for all the four locations.

**Fig. 11:**
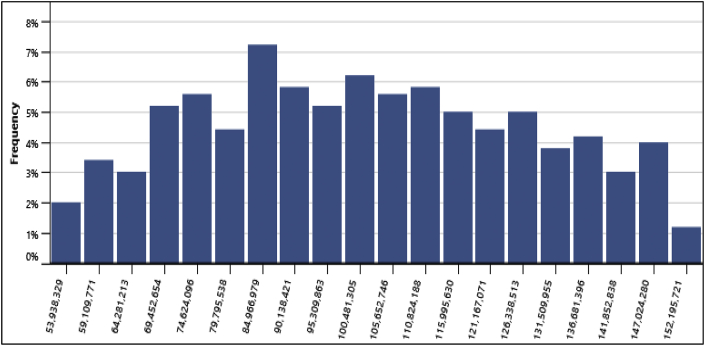
Frequency distribution of the NPV at Anloga.

**Fig. 12:**
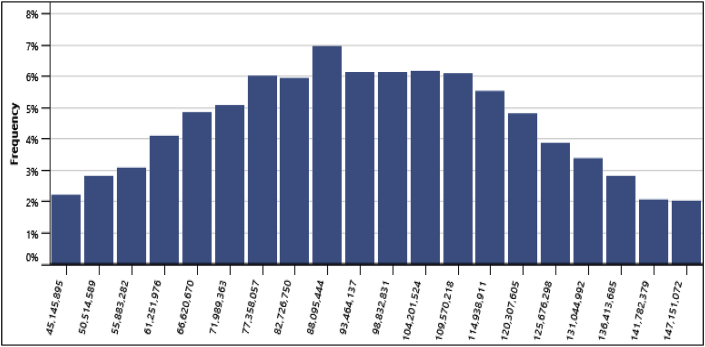
Frequency distribution of the NPV at Atiteti.

**Fig. 13:**
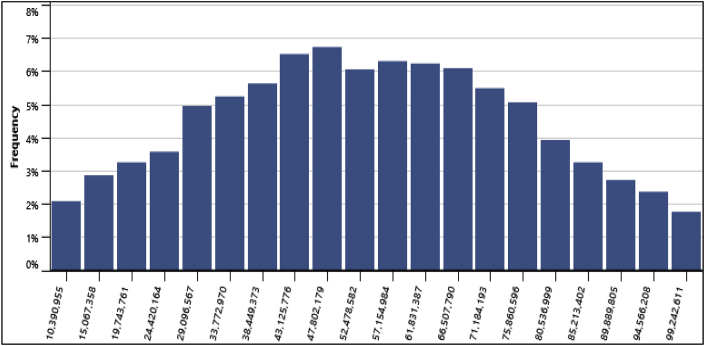
Frequency distribution of the NPV at Sege.

**Fig. 14:**
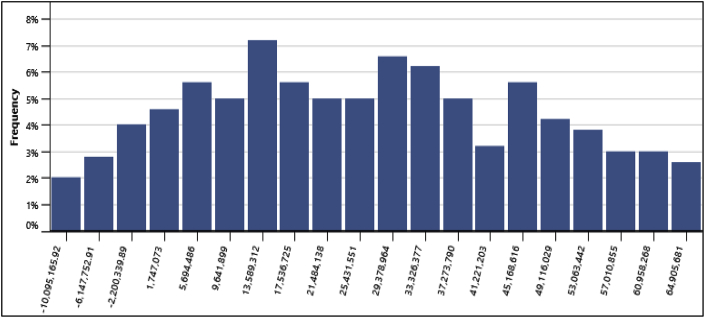
Frequency distribution of the NPV at Denu.

Furthermore, the standard level of risk of 10% was used for conducting risk analysis for this project. At Anloga, the minimum level of confidence for obtaining the NPV was $51,331,453, its maximum level of confidence was $154,790,811 and its median was $99,678,131. This is an indication that the probability of obtaining possible NPV values falling below the maximum level of $154,790,811 was 95% while the probability of obtaining NPV values falling below the minimum level of confidence ($51,331,453) was 5%. Similarly, at Atiteti, the minimum level of confidence for obtaining the NPV was $42,455,052, its maximum level of confidence was $ 149,839,769 and its median was $94,543,670. At Sege, the minimum level of confidence for obtaining the NPV was -$16,675,396, its maximum level of confidence was $52,483,116 and its median was $17,341,322. Finally, at Denu, the minimum level of confidence for obtaining the NPV was -$12,087,407, its maximum level of confidence was $66,880,244 and its median was $26,051,131. In addition, the frequency distribution graphs from the standard risk analysis at the various locations are presented in Fig. 11Fig. 12Fig. 13Fig. 14 respectively. The height of each bar shows the number of occurrences (%) of the NPV values that fall in the range that is specified by the width of the bar. The frequency distribution graphs enable easy assessment of the variability of the financial indicator.

## 5 Conclusion

A complete feasibility analysis was undertaken in this research to determine the possibility of constructing a 50 MW utility-scale wind farm at four locations along Ghana's coast. Technical financial, risk, sensitivity, and GHG emission assessment of the proposed projects were analyzed at the various locations. The assessment was undertaken to aid in attaining a wind energy road map that would serve as information to attract potential wind farm investors from local and international organizations, as well as donor countries towards developing Ghana's wind power potential. This could improve the country's carbon footprint in the fight against global warming which is caused by the utilization of conventional resources for energy. The annual GHG emission reduction if the project is implemented and its potential revenue from carbon trading was also determined. Finally, the financial implications of a potential project, its sensitivity, and risk were also assessed. All the four locations yielded a positive NPV's which is an indication that a potential wind farm project is financially viable at the locations.

However, Anloga and Atiteti yielded favorable equity paybacks which were within the assumed debt term that was considered in this article. The risk analysis revealed that the electricity exported to the grid and the electricity export rate had the greatest impact on the NPV. Considering that the Electricity Exported to the Grid and the Electricity Export Rate (EER) was seen to have the greatest impact on the NPV of the project, any potential investor must pay attention to reducing uncertainties especially, with the EER when negotiating with the utility provider. The capacity of the wind farm can be adjusted with the availability of land and turbine micro-siting. Therefore, this can be done by using Geographical Information Systems to make reasonable site-specific feasibility analyses for future work.

## Author contribution statement

Samuel Sarpong Asamoah: conceived and designed the experiments; performed the experiments; analyzed and interpreted the data; contributed reagents, materials, analysis tools or data; wrote the paper.

Joseph Parbey: analyzed and interpreted the data; contributed reagents, materials, analysis tools or data; wrote the paper.

Isaac Yankey, Alfred Awuah: contributed reagents, materials, analysis tools or data; wrote the paper.

## Funding statement

This research did not receive any specific grant from funding agencies in the public, commercial, or not-for-profit sectors.

## Data availability statement

Data included in article/supp. material/referenced in article.

## Declaration of interest's statement

The authors declare no conflict of interest.

## Nomenclature

$/kWh: Dollars per kilowatt hour
CO_2_: Carbon Dioxide
EPC: Electricity Production Cost
GEDAP: Ghana Energy Development and Access Project
DC: Direct Current
tCO_2_: Tonnes of Carbon Dioxide
GHG: Greenhouse Gas
GW: Gigawatts
IRR: Internal Rate of Return
LCOE: Levelized Cost of Energy
kWh: Kilowatt Hour
m/s: Metres per second
MW: Megawatts
MWh: Megawatt hour
MW: Megawatt power
NPV: Net Present Value
NASA: National Aeronautics and Space Agency (Canada)
REMP: Renewable Energy Master Plan
SO_2_: Sulphur dioxide
SPB: Simple Payback Period
